# An Interesting Case of Lightning Stroke

**Published:** 1905-04

**Authors:** N. C. Mitra

**Affiliations:** Ranchi, Bengal, India


### AN INTERESTING CASE OF LIGHTNING-STROKE.

By N. C. MITRA, M.A., M.B.
Ranchi, Bengal, India.

In the afternoon of a rather hot day in the last week of April it rained and shortly after a tremendous roar of thunder was heard. I was at that time standing on the veranda of my house. It was a deafening sound. It seemed that the lightning had struck close by. Immediately after I detected a smell of sulphur. Out of curiosity, I came out of my house and saw a number of people collected under a tree about one hundred feet distant from my house. They were trying to raise a man who was lying on the ground. I thought the man had fallen to the ground through nervousness and never for a moment surmised that the lightning had struck so close under my nose. I saw the man being brought to a druggist's shop close by. I went there and was told that the man was standing under the tree in question to take shelter from the rain. A few minutes later the tree was struck by the lightning and he fell flat on the ground. I found him suffering intensely from shock with a small, thready pulse. He was complaining of pain over the loins and both the legs. I ordered some brandy mixture for him and went to the post and telegraph office, about sixty feet from the tree, to find out whether the lightning-stroke had been felt there. On entering the post office I noticed a strong sulphurous smell. I then went to the telegraph room which adjoins the postoffice. The signaller gave me a graphic description of the effects of the lightning-stroke on the telegraph machine. He said he was not at work at the time of the occurrence, but was standing close to the instrument. He heard a deafening sound and immediately following he felt as if the whole of the right side of his face was burnt, and through fear he ran out of his room into the adjoining postoffice. Two clerks who were seated close to the entrance into the telegraph room saw, immediately following the thunder, two round burning balls of fire passing out of the telegraph room into the postoffice and then out through the door into the sky. The panes of the glass windows over which the wires passed from the outside into the room were broken into pieces; the copper wires which formed the connecting link between the instrument inside the room and outer wires were found to have melted away; no trace of them could be found. Had the signaller been at work on the instrument he would have been killed on the spot. On further examination, it was found out that the wire connecting the lightning-discharger of the telegraph machine with the ground with the water of an adjoining well had been taken out and through negligence was not put back. It is quite apparent that the return current, on account of imperfect earth connection, was responsible for all the mischief. The telegraph wires passed close by the tree which had been struck. The lightning in the first instance seems to have struck the tree, a small twig of which broke down, but finding the wire close by passed along it to the telegraph office.

From inquiries it was elicited that the patient was a newcomer to the town and happened to be passing along the road when it was raining. He took shelter under the tree and stood with his back against the trunk. In a moment's time he heard the roar, and instantaneously he felt that he was taken off the ground and dashed against it four or five feet distant. He fell flat on the ground. He was first taken to the druggist's shop, and thence to the hospital close by. On admission he complained of intense pain over the loins and the lower extremities. He vomited out several times. The vomited matter consisted of semi-digested rice. He was very uneasy and thought his end was coming on. He passed one loose motion. Later in the evening his friends removed him from the hospital to their lodgings in the town. At about four in the morning I was waked up and asked to see him, as he had passed twenty to twenty-five loose motions, small in quantity but watery in character. I found him very restless and uneasy, tossing on his bed; he had no sleep during the night, his pulse was small and weak, he was perfectly conscious. There had been no more vomiting since. He was still suffering from shock, and wore an anxious appearance. I gave him a sedative astringent mixture. In the morning the watery motions gave place to purely bloody evacuations, and the blood passed was a large quantity. The case was apparently getting worse. I gave him Burroughs Wellcome's Tabloid Hydrastin. It had an admirable effect. After using three tabloids the bloody motions stopped and the patient gradually got better.

This peculiar phenomenon after lightning-stroke does not find mention anywhere, and requires an explanation. The symptoms all point to electrical discharge passing through the lower part of the body, from waist downwards. It is noteworthy that the organs above these escaped altogether. The most satisfactory explanation seems to be that the man was standing with his back against the tree—the parts about the waist were in actual contact with the tree, while the upper part of the body was not touching it. Trees being bad conductors allow lateral discharges; some of these discharges, coming down from the branch struck by the lightning, passed along the trunk through the waist of the patient downwards into the ground. This caused irritation in the bowels, resulting in diarrhea and bloody evacuations and pain in the lower limbs and waist. The greater portion of the electrical discharge passed along the wires into the telegraph office, whilst a feeble one went along the trunk of the tree and the body of the man into the ground, or otherwise the man would have succumbed on the spot, if he had received the full force of the current. The balls of fire witnessed by the postoffice employees passing out of the telegraph office was the very unusual phenomena of *globe lightning.*

